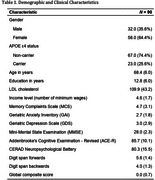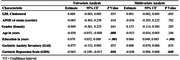# Absence of association between LDL‐cholesterol and cognition outcomes among cognitively healthy older adults from a middle‐income population sample

**DOI:** 10.1002/alz70855_101576

**Published:** 2025-12-27

**Authors:** Mateus Balleiro Bertoldo Garcia, Sabrina Dorta de Oliveira, Cecilia Patricia Popolin, Lucas Nogueira de Carvalho Pelegrini, Ari Alex Ramos, Marcia Regina Cominetti

**Affiliations:** ^1^ Federal University of São Carlos, São Carlos, São Paulo, Brazil; ^2^ Federal University of São Carlos, São Carlos, SP, Brazil; ^3^ Federal University of Sao Carlos, Sao Carlos, SP, Brazil; ^4^ The Global Brain Health Institute, Trinity College Dublin, Dublin, Dublin, Ireland

## Abstract

**Background:**

Recently, higher LDL‐cholesterol levels have been linked to an increased risk of dementia in older adults, raising questions about its potential impact on cognitive function. To explore this connection further, this study examined the association between LDL‐cholesterol and cognition, taking into account demographic, clinical, and genetic variables

**Method:**

We included 90 cognitively healthy older adults (64.4% females) aged 55 and over in this cross‐sectional study. Participants were administered the Addenbrooke's Cognitive Examination ‐ Revised (ACE‐R) and the CERAD Neuropsychological Battery, along with forward and backward digit span tasks. The lack of cognitive impairment was ascertained using the Clinical Dementia Rating (CDR). Linear regression models were fitted to analyze the associations between global cognition composite scores and LDL cholesterol, while simultaneously controlling for APOE ε4 genotype, demographic variables (age, sex, and education), and clinical factors (Geriatric Anxiety Inventory [GAI] and Geriatric Depression Scale [GDS]) in the multivariate analysis.

**Result:**

We found no statistically significant associations between LDL cholesterol and cognitive outcomes in either the univariate (β = 0.000, 95% CI [‐0.003, 0.003], *p* = .935) or multivariate (β = 0.000, 95% CI [‐0.003, 0.003], *p* = .935) models. As expected, in the mutually adjusted model the number of years spent in education were positively associated with cognitive function (β = 0.066, 95% CI [0.048, 0.085], *p* < .001), whereas depression symptoms showed a negative association (β = ‐0.048, 95% CI [‐0.096, 0.000], *p* < .049).

**Conclusion:**

In this study, LDL cholesterol showed no significant impact on cognition. Higher educational attainment emerged as a potential protective factor, highlighting the need for further investigation. Consequently, additional research is required better to understand the relationship between LDL cholesterol and cognitive function.